# Cytotype Regulation Facilitates Repression of Hybrid Dysgenesis by Naturally Occurring *KP* Elements in *Drosophila melanogaster*

**DOI:** 10.1534/g3.116.028597

**Published:** 2016-04-25

**Authors:** Michael J. Simmons, Craig D. Grimes, Cody S. Czora

**Affiliations:** Department of Genetics, Cell Biology, and Development, University of Minnesota, St. Paul, Minnesota 55108-1095

**Keywords:** *P* element, hybrid dysgenesis, piRNA, telomere, ping-pong cycle

## Abstract

*P* elements inserted in the Telomere Associated Sequences (TAS) at the left end of the X chromosome are determiners of cytotype regulation of the entire *P* family of transposons. This regulation is mediated by Piwi-interacting (pi) RNAs derived from the telomeric *P* elements (*TP*s). Because these piRNAs are transmitted maternally, cytotype regulation is manifested as a maternal effect of the *TP*s. When a *TP* is combined with a transgenic *P* element inserted at another locus, this maternal effect is strengthened. However, when certain *TP*s are combined with transgenes that contain the small *P* element known as *KP*, stronger regulation arises from a zygotic effect of the *KP* element. This zygotic effect is observed with transgenic *KP* elements that are structurally intact, as well as with *KP* elements that are fused to an ancillary promoter from the *hsp70* gene. Zygotic regulation by a *KP* element occurs only when a *TP* was present in the maternal germ line, and it is more pronounced when the *TP* was also present in the grand-maternal germ line. However, this regulation does not require zygotic expression of the *TP*. These observations can be explained if maternally transmitted piRNAs from *TP*s enable a polypeptide encoded by *KP* elements to repress *P* element transposition in zygotes that contain a *KP* element. In nature, repression by the KP polypeptide may therefore be facilitated by cytotype-mediating piRNAs.

Hybrid dysgenesis is a syndrome of germ line abnormalities found in the offspring of crosses between different types of *Drosophila* strains (Kidwell *et al.* 1977). These abnormalities include high frequencies of mutation and chromosome breakage and a temperature-dependent form of sterility characterized by the death of germ line cells, a condition termed gonadal dysgenesis (GD). All these traits are caused by the activity of transposable elements. Although different types of transposons may be involved, here we focus on the *P* elements of *Drosophila melanogaster*.

*P*-induced hybrid dysgenesis occurs in the offspring of crosses between paternally contributing (P) and maternally contributing (M) strains (Kidwell *et al.* 1977; Engels 1989). P strains possess *P* elements in their genomes whereas M strains usually do not (Bingham *et al.* 1982). In a dysgenic cross (P male × M female), paternally contributed *P* elements are activated in the germ lines of the offspring. This activation is catalyzed by an 87 kDa enzyme, the P transposase, whose 751 amino acids are encoded by structurally complete members of the *P* element family (Rio 1990). These elements are 2907 bp long and are demarcated by 31 bp long inverted terminal repeats (O’Hare and Rubin 1983), which are the substrates for transposase action. Structurally incomplete *P* elements cannot produce the transposase, but they can be mobilized by it if they possess the inverted terminal repeats. In nature, *P* element activity is restricted to the germ line because of the way in which the *P* element’s primary RNA is spliced. All three of the *P* element’s introns can be removed in the germ line, but in the soma, the last of the introns remains (Laski *et al.* 1986). This last intron contains a stop codon, which terminates translation prematurely and prevents the transposase from being made.

In the germ line, *P* element activity can be repressed by different regulatory factors. Some are *P*-encoded polypeptides with substantial homology to the P transposase, but without its catalytic activity. One of these repressors is the 66 kDa polypeptide translated from incompletely spliced *P* RNA (Robertson and Engels 1989; Misra and Rio 1990; Misra *et al.* 1993; Gloor *et al.* 1993; Andrews and Gloor 1995). Other repressor polypeptides are translated from the transcripts of structurally incomplete *P* elements that are too small to encode the transposase. The best known of these small elements is called *KP* because it was discovered in flies from the Russian city of Krasnodar (Black *et al.* 1987; Jackson *et al.* 1988). *KP* elements are missing base pairs 809–2561 in the canonical *P* element sequence. Consequently, they encode a polypeptide of only 207 amino acids, 199 of which are identical to the beginning of the transposase. The *KP* polypeptide binds to *P* elements and represses transposition (Lee *et al.* 1996, 1998); it also appears to repress *P* element expression (Lemaitre *et al.* 1993) and to play a role in heterochromatin formation (Bushey and Locke 2004; Sameny and Locke 2011). Surveys have shown that *KP* elements are widespread in natural populations (Boussy *et al.* 1988; Itoh and Boussy 2002; Itoh *et al.* 2007; C. R. Preston and W. R. Engels, personal communication), a finding consistent with the idea that the KP repressor confers a selective advantage on flies that can make it.

Germ line *P* element activity is also repressed by a maternally transmitted condition called the P cytotype, a hallmark of all P strains (Engels 1979, 1989). Repression by the P cytotype involves small RNAs generated from *P* elements inserted at special loci in the genome. These RNAs, called Piwi-interacting (or pi) RNAs because they associate with members of the Piwi family of proteins, are deposited in the oocyte to protect the future offspring from the untoward effects of rampant *P* transposition (Brennecke *et al.* 2008). On account of this maternal endowment, flies from crosses between P females and M males show much lower levels of dysgenesis than flies from reciprocal crosses; likewise, flies from crosses between P females and P males seldom show dysgenic traits.

Genetic studies indicate that *P* elements inserted in the TAS at the left end of the X chromosome are determiners of the P cytotype (Ronsseray *et al.* 1991, 1993, 1996, 1998; Marin *et al.* 2000; Stuart *et al.* 2002; Simmons *et al.* 2004; Niemi *et al.* 2004; Jensen *et al.* 2008). The regulatory power of these elements is enhanced when other *P* elements at nontelomeric loci are added to the maternal genotype, even though the latter have no intrinsic ability to repress dysgenesis (Simmons *et al.* 2007, 2012, 2014). This synergism is thought to result from a process that amplifies the pool of *P*-specific piRNAs. In brief, antisense piRNAs derived from the telomeric *P* elements target and cleave long sense RNAs derived from the nontelomeric *P* elements, generating sense piRNAs that can target antisense transcripts from the telomeric elements. With repetition, this process—called the ping-pong cycle (Aravin *et al.* 2007; Gunawardane *et al.* 2007)—generates a large population of piRNAs, which can then be transmitted maternally to minimize dysgenesis in the next generation. These piRNAs undercut transposase synthesis either by degrading *P* mRNAs or by preventing their translation (Jensen *et al.* 2008). They may also repress transposition by influencing the structure of chromatin around *P* elements (Josse *et al.* 2007).

A recent study has revealed another dimension of piRNA-based cytotype regulation. Telomeric *P* elements acting maternally facilitate repression of dysgenesis by the *KP* element acting zygotically (Simmons *et al.* 2015). This phenomenon was discovered by combining different X-linked telomeric *P* elements with *hobo*-element transgenes designed to express the KP polypeptide. The *KP* elements within these *hobo* transgenes were terminally truncated to preclude mobilization by the P transposase. They were also supplied with an ancillary promoter (from the *Drosophila hsp70* gene). By themselves, these *H*(*hsp*/*KP*) transgenes could not repress dysgenesis. However, when combined with a cytotype-determining telomeric *P* element (*TP*), they could. This emergent property was manifested as a zygotic effect in the progeny of test crosses to induce dysgenesis—that is, it was seen only in the progeny that inherited the *H*(*hsp*/*KP*) transgene, even if those progeny had not inherited the *TP*. These observations suggest that *TP*-derived piRNAs enable transgene-encoded KP polypeptides to repress hybrid dysgenesis.

In this paper, we explore this unanticipated connection between the piRNA and polypeptide mechanisms of *P* element regulation further by determining if cytotype-determining *TP*s have the same effect on naturally occurring *KP* elements as on *hsp*/*KP* fusions. Specifically, we show that a *TP* enables an intact *KP* element situated within a transgenic genomic fragment to repress GD through a zygotic effect. This finding demonstrates that cytotype regulation empowers naturally occurring *KP* elements that have not been augmented with an ancillary promoter to repress *P* element transposition, presumably by facilitating the action of the KP polypeptide.

## Materials and Methods

### Drosophila stocks and husbandry

Information on genes and mutant alleles can be found on the Flybase website, in Lindsley and Zimm (1992), or in references cited in the text. The *P* elements *TP5* and *TP6* were isolated in the telomere of the left arm of X chromosomes from the wild-type strains ν_6_ and Mt. Carmel, respectively (Stuart *et al.* 2002). The *KP* element known as *KP1* was isolated in cytological region 2F in the X chromosome from the wild-type strain Sexi.1 (Rasmussen *et al.* 1993). Maps of the *TP5*, *TP6*, and *KP* elements are presented in Jensen *et al.* (2008) and in Simmons *et al.* (2015). The standard M strain used in the experiments carried the markers *y* and *w^67c23^* (hereafter, denoted simply as *w*) and is devoid of *P* elements. The standard P strain was Harwich *y w*, which was previously derived from the Harwich *w* strain of Kidwell *et al.* (1977) by incorporating the *y* and *w^67c23^* markers (Simmons *et al.* 2012). Harwich *y w* carries a plethora of *P* elements, including some that encode the P transposase; however, it does not carry any *KP* elements (Simmons *et al.* 2015). *Drosophila* cultures were reared in vials on a standard cornmeal-molasses-yeast medium. Stock cultures were incubated at 18–21°. Strains are available upon request.

### H(hsp/KP) transgenic stocks

The *H*(*hsp*/*KP*) transgene contains a *KP* element truncated before base pair 39 in the 5′ end and after base pair 2871 in the 3′ end of the canonical *P* element sequence (Simmons *et al.* 2002); the 5′ end includes the native *P* element promoter. A cassette containing the promoter from *D. melanogaster*’s *hsp70* gene is situated immediately upstream of the *KP* element. This transgene was constructed from the pHawN *hobo*-element vector, which carries the eye-coloring mini-*white* gene as a marker. It was introduced into *y w Drosophila* by injection into embryos along with pHBL1, a plasmid that encodes the hobo transposase (Calvi and Gelbart 1993). Among the transgenic stocks that were obtained, we used *H*(*hsp*/*KP*)*7*, which carries two loosely linked insertions of the transgene on chromosome 2. When combined with the telomeric *P* elements *TP5* or *NA* (Marin *et al.* 2000), the transgenes in this stock enhance repression of dysgenesis through zygotic effects; when combined with the telomeric *P* element *TP6*, they enhance repression mainly through maternal effects (Simmons *et al.* 2015).

### Cloning of KP1 and creation of H(w^+^, KP1) transgenic stocks

Genomic DNA was isolated from 40 flies carrying the X-linked *KP* element known as *KP1* (Rasmussen *et al.* 1993), which was the only *P* element in these flies. This DNA was digested with *Eco*RI and then recombined with the λZapII bacteriophage vector purchased from Stratagene. Recombinant phages were plated on lawns of XL1 *Escherichia coli* cells and the resulting plaques were screened for the *KP1* element by hybridization with a ^32^P-labeled probe, produced by random priming from a PCR-amplified complete *P* element. A *KP1*-containing phage clone was isolated and the plasmid cassette (pBluescript + *Eco*RI genomic fragment) within the phage vector was obtained by *in vivo* excision. The cloned *Eco*RI fragment in this plasmid was transferred into another plasmid (pMartini), which has *Not*I sites flanking the *Eco*RI cloning site. After digestion with *Not*I, the *KP1*-containing genomic fragment was inserted into the unique *Not*I site of the *hobo* transformation vector pHawN. The resulting construct, denoted *H*(*w*^+^, *KP1*), was injected into *y w Drosophila* embryos along with pHBL1 to obtain transgenic stocks, and insertions were localized to specific chromosomes by segregation against dominant markers. The *KP1* clone was analyzed by DNA sequencing using *P* element-specific primers, and the genomic position of the *KP1* element was determined by comparing the sequence data to the *D. melanogaster* genome using the BLAST analytic.

### Assay for GD

GD was induced by mass mating test females to males from the Harwich *y w* P strain at 21°. After 3 d, the mated females were individually transferred to fresh cultures, which were reared at 29°. On day 11, the offspring that had hatched from each culture were transferred to a holding vial, where they matured for 2 d at 21°. The frequency of GD was determined by squashing samples of the females among these offspring between two glass slides to see if they had any eggs. Females with GD do not produce eggs. Green food coloring was placed between the slides to facilitate the scoring of this egg-less phenotype. When the test crosses segregated different types of females, each type was scored separately, with a maximum of 20 females per type.

The females that were used in the test crosses to Harwich *y w* males were obtained from different types of crosses, which are described in *Results*. These initial crosses were incubated at 25°.

### Statistical analyses

The frequency of GD was calculated independently for each type of female in each test vial. Unweighted average frequencies and empirical standard errors (SE) among all the vials in a test group were then computed for each type. Statistical differences between averages were evaluated by performing *t* or *z* tests. GD frequencies were also computed by pooling data across types of females in each vial. Differences between pooled groups (females carrying a transgene *vs.* females not carrying it) were summarized by the number of vials in which one group had less GD than the other group. This “comparison score” between the two groups was evaluated using the nonparametric sign test.

### Data availability

The authors state that all data necessary for confirming the conclusions presented in the article are represented fully within the article.

## Results

### The H(w^+^, KP1) hobo transgene contains an intact KP element

Individual *KP* elements had previously been isolated in X chromosomes derived from a wild-type strain known as Sexi (Kidwell 1985). One of these elements, denoted *KP1*, was localized to region 2F of the X chromosome, and genetic tests had shown that it could repress hybrid dysgenesis in some assays (Rasmussen *et al.* 1993). We cloned this *KP* element from a recombinant DNA library and used it to create a *hobo* transgene marked with the mini-white gene (here symbolized *w*^+^), which served as a convenient visible marker for subsequent genetic analyses. The transgene is denoted *H*(*w*^+^, *KP1*).

The *KP1* element within *H*(*w*^+^, *KP1*) is situated 1.3 kb from the distal end of a 7 kb *Eco*RI fragment derived from cytological region 2F5 in the X chromosome of the Sexi strain. Sequence analysis showed that the element is intact, that it is oriented 5′ to 3′ from distal to proximal in the cloned fragment, and that the 8 bp target site duplication created when the element inserted into this region begins at nucleotide 2,197,975 of the reference *D. melanogaster* genome sequence. This insertion site is located well within the long intron of the *pole hole* (*phl*) gene.

### H(w^+^, KP1) transgenes do not repress Harwich-induced GD

We first assessed whether or not the *H*(*w*^+^, *KP1*) transgenes in two different stocks, denoted C and J, have an intrinsic ability to repress GD induced by the strong P strain Harwich *y w*. This assessment was accomplished by crossing *y w*; *H*(*w*^+^, *KP1*)/+ females to Harwich *y w* males and scoring the incidence of GD in their two types of daughters—those carrying the *H*(*w*^+^, *KP1*) transgene (identified by their colored eyes) and those not carrying it (identified by their white eyes). The *y w*; *H*(*w*^+^, *KP1*)/+ females for these tests were obtained by crossing homozygous *y w*; *H*(*w*^+^, *KP1*) females to *y w* males from an M strain. In stock C, the *H*(*w*^+^, *KP1*) transgene is located on chromosome 2; in stock J, two loosely linked insertions of this transgene are located on chromosome 3. Along with these stocks, we also tested a stock that carries two insertions of *H*(*hsp*/*KP*), a transgene that has been studied previously (Simmons *et al.* 2015).

Table 1 summarizes the results of these tests for repression of GD by the two kinds of *KP* transgenes. All the testcross offspring that did not inherit a transgene were dysgenic. Among the offspring that did inherit a transgene, only those from the *H*(*hsp*/*KP*)*7* stock showed a statistically significant reduction in GD frequency (*P* < 0.05), and it was slight. Thus, the *H*(*w*^+^, *KP1*) and *H*(*hsp*/*KP*) transgenes have, at best, a very weak intrinsic ability to repress GD induced by the Harwich *y w* P strain.

**Table 1:** Intrinsic inability of *KP* transgenes to repress gonadal dysgenesis

		Transgene Absent		Transgene Present	
Transgenic Stock	No. of Vials	No. of Flies	%GD ± SE*^a^*	No. of Flies	%GD ± SE*^a^*
*H*(*hsp*/*KP*)*7**^b^*	24	316	100 ± 0	365	94.8 ± 1.7
*H*(*w*^+^, *KP1*)*C*	25	279	100 ± 0	269	100 ± 0
*H*(*w*^+^, *KP1*)*J**^b^*	25	180	100 ± 0	367	98.6 ± 0.6

GD, gonadal dysgenesis.

a Unweighted average percentage GD ± SE.

b Two loosely linked insertions of the transgene are present in these stocks.

### The P cytotype enables the KP transgenes to repress GD

We next investigated whether or not the P cytotype could enhance the ability of the two kinds of *KP* transgenes to repress GD. In these tests, the P cytotype was determined by *P* elements inserted in the TAS of the XL telomere. In one experiment, the telomeric *P* element was *TP5*; in another, it was *TP6*. These two elements are similar in size and are inserted in the same site within one of the repeating DNA units within the TAS (Stuart *et al.* 2002). The *TP5* element shares less DNA sequence with the *KP* element than the *TP6* element does. Previous work has shown that both *TP5* and *TP6* interact synergistically with the transgenes in the *H*(*hsp*/*KP*)*7* stock to repress GD, and with the *H*(*hsp*/*KP*) transgenes in other stocks as well (Simmons *et al.* 2015). With *TP5*, this synergism is due mainly to a zygotic effect of the transgenes, whereas with *TP6* it is due mainly to a maternal effect of the *TP6-H*(*hsp*/*KP*)*7* transgene combination. Thus, the *H*(*hsp*/*KP*)*7* transgenes served as positive controls in these experiments.

The experiments began by reciprocally crossing flies from the two *TP* and the three transgenic *KP* strains. The transgenic strains were homozygous for the markers *y* and *w*, which are tightly linked to the XL telomere; the *TP* strains were homozygous for the *w* marker only. The crosses using *TP* females are denoted with the letter A; those using transgenic females are denoted with the letter B. The *TP y*^+^ *w*/*y w*; *H*(*KP*)/+ F_1_ females from these reciprocal crosses were then mated to Harwich *y w* males, and their F_2_ daughters with different body and eye color phenotypes were scored for GD. Daughters with wild-type body color carried the *TP*, and daughters with colored eyes carried the *KP* transgene. This design allowed us to determine if repression of GD involved maternal or zygotic effects of the telomeric and transgenic *P* elements. Because the tested F_1_ females were obtained from reciprocal crosses, we could also determine if repression depended on the parental origin of the telomeric *P* element. Each of the experiments also included controls without transgenes to determine the intrinsic repression abilities of the telomeric *P* elements.

Table 2 summarizes the results of the experiment involving *TP5*. A solitary *TP5* element derived maternally from cross A repressed GD moderately (63.6% GD overall), but when paternally derived from cross B, it repressed GD very weakly (92.8% GD overall). Thus, as expected from previous studies (Simmons *et al.* 2012, 2014, 2015), the parental origin of the cytotype-determining *TP5* element has a significant effect on repression ability. However, among the F_2_ flies that were scored for GD, there was no significant difference between those that inherited *TP5* and those that did not. Thus, as previously reported (Thorp *et al.* 2009), repression of GD in these kinds of control crosses is mediated by a strictly maternal effect of the *TP5* element.

**Table 2:** Repression of gonadal dysgenesis in the daughters of *TP5 y^+^ w*/*y w*; *hobo*/+ females from reciprocal crosses between *TP5 y^+^ w* and *y w*; *hobo* strains

Transgenic Stock	Cross	No. of Vials	Neither*^a^*		Transgene Only*^a^*		*TP5* Only*^a^*		Both*^a^*		Transgene Absent*^b^*		Transgene Present*^b^*		Comparison Score*^d^*
			No. of Flies	%GD ± SE*^c^*	No. of Flies	%GD ± SE*^c^*	No. of Flies	%GD ± SE*^c^*	No. of Flies	%GD ± SE*^c^*	No. of Flies	%GD ± SE*^c^*	No. of Flies	%GD ± SE*^c^*	
None	A	23	301	68.2 ± 5.3			320	59.3 ± 6.0			621	63.6 ± 5.5			
*H*(*hsp*/*KP*)*7*	A	14	55	54.0 ± 9.4	78	25.8 ± 4.8	73	69.7 ± 8.1	79	15.6 ± 5.4	128	62.3 ± 6.5	157	21.3 ± 4.2	13*
*H*(*w*^+^, *KP1*)*C*	A	26	127	79.2 ± 5.2	136	43.4 ± 6.6	152	72.1 ± 5.1	115	28.6 ± 3.7	279	76.2 ± 3.4	251	37.7 ± 4.7	26*
*H*(*w*^+^, *KP1*)*J*	A	25	124	91.6 ± 4.3	225	74.2 ± 3.5	125	91.6 ± 3.5	271	74.8 ± 3.3	249	91.8 ± 2.8	496	73.8 ± 3.0	20*
None	B	24	231	94.2 ± 1.5			259	91.1 ± 1.9			490	92.8 ± 1.2			
*H*(*hsp*/*KP*)*7*	B	22	97	97.6 ± 1.7	120	92.3 ± 3.1	98	97.3 ± 1.9	143	84.8 ± 3.6	195	97.2 ± 1.3	263	87.9 ± 2.5	15
*H*(*w*^+^, *KP1*)*C*	B	25	131	98.4 ± 1.1	156	84.1 ± 4.8	159	90.6 ± 3.9	118	81.7 ± 5.3	290	94.5 ± 2.1	274	84.0 ± 4.2	15
*H*(*w*^+^, *KP1*)*J*	B	20	68	94.3 ± 3.1	187	91.8 ± 3.0	88	94.9 ± 2.8	201	88.0 ± 3.0	156	93.6 ± 3.0	388	89.2 ± 2.6	12

* Indicates numbers that are statistically significant by the sign test. GD, gonadal dysgenesis; A, crosses using *TP* females; B, crosses using transgenic females.

a Two factors—the telomeric element (*TP5*) and the *hobo* transgene—segregated in the test crosses, giving rise to four genotypic classes in the F_2_. The headings indicate which of these two factors were present in the females that were scored for GD.

b The four categories of data have been pooled into two categories to assess the effect of the transgene in the F_2_ females that were scored for GD.

c Unweighted average percentage GD ± SE.

d Number of vials in which the F_2_ females carrying the transgene had a lower GD frequency than the females not carrying it.

When a *KP* transgene was present in the tested females from cross A, repression of GD was markedly enhanced, but only among the offspring that inherited the transgene. We observed this zygotic effect with all three of the stocks that were tested, but most strongly with *H*(*hsp*/*KP*)*7*. The frequency of GD among the F_2_ females that inherited an *H*(*hsp*/*KP*) transgene was 21.3%, whereas among the F_2_ females that did not, it was 62.3%. As judged by the comparison score computed from the GD frequencies within replicate test cultures, this difference is highly significant (*P* < 0.01). Thus, when combined with a *TP5* element derived from cross A, the *H*(*hsp*/*KP*) transgenes strongly repress GD through a zygotic effect in the F_2_ testcross offspring.

The *H*(w^+^, *KP1*) transgenes also repressed GD through zygotic effects in cross A. For stock C, 37.7% of the F_2_ flies that inherited the *H*(w^+^, *KP1*) transgene were dsygenic, compared to 76.2% of the F_2_ flies that did not inherit it; for stock J, the corresponding frequencies were 73.6% and 91.8%. For each stock, these differences are statistically significant (*P* < 0.05). Thus, as with the *H*(*hsp*/*KP*) transgenes, a *TP5* element derived from Cross A enables the *H*(w^+^, *KP1*) transgenes to repress GD through a zygotic effect.

In cross B, the offspring that carried either a *H*(*hsp*/*KP*) or a *H*(*w*^+^, *KP1*) transgene had slightly lower frequencies of GD than those that did not carry a transgene. This pattern suggests that, as in cross A, the *H*(*hsp*/*KP*) and *H*(*w*^+^, *KP1*) transgenes repressed GD through zygotic effects. However, none of these effects were statistically significant. The absence of significant repression in cross B underscores the importance of the parental origin of the cytotype-determining *TP5* element.

Table 3 summarizes the results of the experiment involving *TP6*. Unlike *TP5*, a solitary *TP6* element had little ability to repress GD, even in the tests from cross A (97.7% GD). However, when *TP6* was combined with a *KP* transgene through either cross A or cross B, GD was repressed in the offspring from some of the testcrosses. The strongest repression was seen in the F_2_ flies derived from the *TP6 y*^+^ *w*/*y w*; *H*(*hsp*/*KP*)*7*/+ F_1_ females from cross A. In this case, only 18.5% of the F_2_ flies that inherited an *H*(*hsp*/*KP*) transgene were dysgenic, compared to 74.9% of those that did not. When compared to the control with *TP6* alone (97.7% GD), these frequencies indicate that in cross A GD was repressed through a combination of weak maternal effects of the *TP6-H*(*hsp*/*KP*)*7* F_1_ genotype and strong zygotic effects of the *H*(*hsp*/*KP*)*7* transgenes. In cross B, 35.7% of the F_2_ flies that inherited an *H*(*hsp*/*KP*)*7* transgene were dysgenic, compared to 98.3% of those that did not. Thus, in cross B, GD was repressed exclusively by the zygotic effects of the *H*(*hsp*/*KP*) transgenes.

**Table 3:** Repression of gonadal dysgenesis in the daughters of *TP6 y^+^ w*/*y w*; *hobo*/+ females from reciprocal crosses between *TP6 y^+^ w* and *y w*; *hobo* strains

			Neither*^a^*		Transgene Only*^a^*		*TP6* Only*^a^*		Both*^a^*		Transgene Absent*^b^*		Transgene Present*^b^*		
Transgenic Stock	Cross	No. of Vials	No. of Flies	%GD ± SE*^c^*	No. of Flies	%GD ± SE*^c^*	No. of Flies	%GD ± SE*^c^*	No. of Flies	%GD ± SE*^c^*	No. of Flies	%GD ± SE*^c^*	No. of Flies	%GD ± SE*^c^*	Comparison Score*^d^*
None	A	25	260	97.8 ± 1.3			246	98.0 ± 0.9			506	97.7 ± 1.1			
*H*(*hsp*/*KP*)*7*	A	15	67	73.8 ± 6.7	66	25.7 ± 9.3	60	75.8 ± 7.6	49	11.4 ± 5.6	127	74.9 ± 5.1	115	18.5 ± 6.0	15*
*H*(*w*^+^, *KP1*)*C*	A	20	134	95.6 ± 2.3	116	60.4 ± 4.0	131	95.7 ± 2.0	92	60.8 ± 6.7	265	96.0 ± 1.5	208	58.9 ± 3.5	20*
*H*(*w*^+^, *KP1*)*J*	A	27	86	99.4 ± 0.6	210	75.4 ± 3.9	68	97.2 ± 1.9	193	72.4 ± 4.9	154	98.5 ± 0.8	403	74.3 ± 3.5	24*
None	B	19	173	99.1 ± 0.9			170	98.2 ± 1.2			343	98.7 ± 1.0			
*H*(*hsp*/*KP*)*7*	B	13	58	100 ± 0	53	36.8 ± 9.1	49	96.2 ± 2.8	68	30.3 ± 7.0	107	98.3 ± 1.1	116	35.7 ± 5.6	13*
*H*(*w*^+^, *KP1*)*C*	B	26	185	98.4 ± 1.6	137	91.9 ± 3.2	148	100 ± 0	128	92.1 ± 2.3	333	99.0 ± 1.0	265	91.6 ± 2.1	12
*H*(*w*^+^, *KP1*)*J*	B	13	24	100 ± 0	82	86.3 ± 3.9	25	100 ± 0	68	93.5 ± 2.9	49	100 ± 0	150	88.7 ± 2.9	10

* Indicates numbers that are statistically significant by the sign test. GD, gonadal dysgenesis; A, crosses using *TP* females; B, crosses using transgenic females.

a Two factors—the telomeric element (*TP6*) and the *hobo* transgene—segregated in the test crosses, giving rise to four genotypic classes in the F_2_. The headings indicate which of these two factors were present in the females that were scored for GD.

b The four categories of data have been pooled into two categories to assess the effect of the transgene in the F_2_ females that were scored for GD.

c Unweighted average percentage GD ± SE.

d Number of vials in which the F_2_ females carrying the transgene had a lower GD frequency than the females not carrying it.

When combined with *TP6*, the *H*(*w*^+^, *KP1*) transgenes from stocks C and J repressed GD through zygotic effects only: moderately in cross A and weakly in cross B. By the sign test, only the effects in cross A were statistically significant (*P* < 0.05). Thus, regulation by *TP6* and the *H*(*w*^+^, *KP1*) transgenes resembles regulation by *TP5* and either type of transgene.

## Discussion

For many years after their discovery, *P* elements were thought to be regulated exclusively by *P*-encoded polypeptides, including the 66 kDa repressor produced by complete *P* elements and the smaller repressor produced by *KP* elements (Rio 1990). This idea was supported by considerable evidence, but it failed to account for the maternal inheritance of cytotype that was seen in all the classic genetic analyses. Cytotype regulation is now understood to be mediated by piRNAs rather than by repressor polypeptides (Brennecke *et al.* 2008; Jensen *et al.* 2008).

*P*-specific piRNAs are generated by *P* elements inserted in the TAS of chromosome XL. These RNAs can be deposited in oocytes, where they provide a defense against *P* element activity in future embryos. Biochemical and genomic analyses have suggested that interactions between these primary piRNAs and long RNA molecules transcribed from transposons at other loci boost overall piRNA abundance. This repetitive process—called the ping-pong cycle—can provide a robust defense against *P* element activity in the next generation (Brennecke *et al.* 2008). Genetic analyses with combinations of telomeric and nontelomeric *P* elements support this idea. The nontelomeric *P* elements have no regulatory abilities of their own, but they can significantly enhance repression of dysgenesis by the telomeric *P* elements (Simmons *et al.* 2007). A key finding is that the enhanced repression is manifested in all the progeny of a test cross, even those that do not inherit any *P* elements (Simmons *et al.* 2012). Enhanced repression is therefore manifested as a strictly maternal effect, which at the molecular level implies that the amplified piRNA pool (or the raw material to produce it) is deposited into oocytes regardless of their genotype.

Genetic analysis of specific combinations of telomeric and nontelomeric *P* elements indicates that the ability to repress hybrid dysgenesis depends on the amount of DNA sequence shared by the participating elements, presumably by influencing the efficacy of ping-pong cycling (Simmons *et al.* 2012; Jessen *et al.* 2013). Too little shared sequence may prevent the cycle from sustaining itself, but extensive shared sequence may also impair the cycle by allowing the formation of long double-stranded RNAs, which may be shunted into a pathway that does not produce piRNAs. Ping-pong cycling may also be influenced by the efficiency of RNA transport from the nucleus into the nuage, the perinuclear organelle in which piRNA production apparently occurs.

Though they are small, *KP* elements appear to have sufficient sequence overlap with the telomeric element *TP6* to sustain ping-pong cycling and enhance cytotype regulation. Females that carried *TP6* and an *H*(*hsp*/*KP*) transgene moderately repressed GD in daughters that inherited neither *TP6* nor *H*(*hsp*/*KP*) (Table 3; Simmons *et al.* 2015). Repression through this strictly maternal effect presumably reflects the transmission of *P*-specific piRNAs that were amplified by ping-pong cycling in the mother’s germ line. However, intact *KP* elements within *H*(*w*^+^, *KP1*) transgenes did not enhance maternal regulation by *TP6*. This shortcoming may be due to differences in *KP* expression in the two types of transgenic stocks.

When either *H*(*hsp*/*KP*) or *H*(*w*^+^, *KP1*) transgenes were combined with the telomeric element *TP5*, enhanced repression of dysgenesis was seen only in flies that carried a transgene—that is, it was manifested as a strictly zygotic effect of the transgene rather than as a maternal effect of the *TP5* and *KP* elements in the mother’s genotype. The absence of a maternal effect suggests that *KP* has insufficient sequence overlap with *TP5* to sustain ping-pong amplification of *P*-specific piRNAs. A maternal effect was also not observed in tests where *H*(*hsp*/*KP*) transgenes were combined with *NA*, a telomeric *P* element that has even less sequence overlap with *KP* than *TP5* does (Simmons *et al.* 2015).

Our data show that cytotype-determining *TP*s enabled *H*(*hsp*/*KP*) and *H*(*w*^+^, *KP1*) transgenes to repress GD through a zygotic effect in testcross offspring that inherited the transgene, even those that did not inherit the telomeric *P* element. This zygotic repression by the transgenic *KP* element is an emergent property not seen with other transgenic *P* elements, and it clearly depends on the P cytotype. We see it strongly in offspring derived from cross A, which have an unbroken maternal lineage of cytotype regulation, but less so in offspring from cross B, which do not. We also see this repression no matter which telomeric *P* element—*TP5*, *TP6*, or *NA*—determines the P cytotype (Simmons *et al.* 2015), although the repression is most pronounced with *TP5* and *NA*, which share less sequence with *KP* than *TP6* does. Zygotic repression by the *KP* element depends on the P cytotype, but it does not require a telomeric *P* element to be present in the females at risk for dysgenesis. Nor does it require the *KP* element to have been associated with a telomeric *P* element in the maternal germ line; *KP* elements derived paternally from a Harwich *y w* strain can repress GD in the female offspring of testcrosses, as long as a telomeric element was present in their mothers (Simmons *et al.* 2015).

All these observations indicate that cytotype regulation mediated by maternally transmitted piRNAs enables *KP* elements to repress dysgenesis through the zygotic expression of a *KP*-derived factor, presumably the KP repressor polypeptide. Of course, it is formally possible that the zygotic effect of the *KP* element is mediated by a *KP*-derived RNA, but the overall pattern of results with different *TP*s and different *KP* transgenes suggests otherwise (Simmons *et al.* 2015). In addition, the repressor function of the KP polypeptide has been well documented *in vitro* (Lee *et al.* 1996, 1998). The mechanism by which maternally transmitted piRNAs derived from a telomeric *P* element enable the expression and/or action of the KP repressor polypeptide is unknown. One possibility is that the piRNAs enhance the ability of the KP polypeptide to bind to *P* elements throughout the genome and repress their movement. In fission yeast, piRNAs appear to be involved in chromatin organization (Grewal 2010), and in *Drosophila*, there is some evidence that they play a role in heterochromatin formation (Josse *et al.* 2007).

The peculiar way in which piRNAs facilitate repression of hybrid dysgenesis by *KP* elements indicates that transposon regulation involves more than the simple depletion or destruction of transposase-encoding mRNAs, and the finding that intact *KP* elements, as well as *hsp*/*KP* fusions, respond to this facilitation indicates that piRNA-repressor polypeptide interactions may be important for transposon regulation in natural populations.
